# Computational Approaches for Protein–DNA/RNA Complex Modeling for Cryo‐EM Maps

**DOI:** 10.1002/cpz1.70409

**Published:** 2026-08-12

**Authors:** Anika Jain, Kefan Cao, Daisuke Kihara

**Affiliations:** ^1^ Department of Biological Sciences Purdue University West Lafayette Indiana; ^2^ Department of Computer Science Purdue University West Lafayette Indiana

**Keywords:** cryo‐EM, deep learning, nucleic acid, protein, structure modeling

## Abstract

Cryogenic electron microscopy (cryo‐EM) has become a key method in structural biology for determining macromolecular structures. Numerous computational tools have been developed to build atomic models from cryo‐EM density maps. However, relatively few tools are available for modeling protein–nucleic acid complexes. Here, we describe how to use two such methods developed by our group, ComplexModeler and CryoZeta, with a focus on modeling protein–nucleic acid complexes. Both tools are available through the EMSuite web server, a freely accessible platform that hosts multiple methods for cryo‐EM structure modeling and validation. ComplexModeler integrates DiffModeler and CryoREAD to construct protein–DNA/RNA complex structures at resolutions of up to 5 Å. DiffModeler employs a diffusion model for backbone tracing, followed by fitting AlphaFold2‐predicted protein structures into the traced backbone. CryoREAD identifies nucleotide components (phosphate, sugar, and base), constructs the backbone, assigns sequences, and builds full atomic models of DNA/RNA chains. CryoZeta uses a diffusion‐based generative model that integrates sequence‐based structure prediction with cryo‐EM density features to generate accurate models of proteins, nucleic acids, and their complexes. This article describes how to use these two tools on the EMSuite web server through two modeling examples. © 2026 The Author(s). *Current Protocols* published by Wiley Periodicals LLC.

**Basic Protocol 1**: Protein–nucleic acid structure modeling using ComplexModeler on the EMSuite server

**Basic Protocol 2**: Protein–nucleic acid structure modeling using CryoZeta on the EMSuite server

## INTRODUCTION

Interactions of proteins with DNA or RNA are important for cellular function. Determining the three‐dimensional (3D) structures of these interacting macromolecules is an important facet of uncovering the underlying mechanisms of their biological function. One such experimental method that has gained popularity in recent years for structure determination is cryogenic electron microscopy (cryo‐EM).

Modeling structures into cryo‐EM density maps requires computational methods. While initial algorithms were conventional tracing methods (Chen et al., 2016; Terashi & Kihara, 2018), deep learning approaches have proven highly effective (Farheen et al., 2025; S. Li et al., 2025). Early methods primarily focused on protein modeling (Giri & Cheng, 2024; Pfab et al., 2021; Terashi et al., 2024a), while more recent approaches have also addressed modeling of DNA or RNA chains alone (T. Li et al., 2024,  2025; Wang et al., 2023). However, relatively few methods target protein–DNA/RNA complexes: EMInfo identifies protein secondary structures and nucleic acid regions via map segmentation but does not construct atomic models (Cao et al., 2025). DEMO‐EMol extends this approach by generating full atomic models through segmentation followed by iterative placement of pre‐built chain structures and global optimization, though it relies on the accuracy of the initial chain models (Zhang et al., 2025). DeepTracer2.0 detects protein and nucleic acid regions and models them separately, constructing protein backbones from Cα atoms and nucleotide backbones from phosphate atoms, before combining them into a final complex, which may limit accurate modeling of intermolecular interactions (Nakamura et al., 2023). ModelAngelo constructs atomic models of protein and DNA/RNA complexes using a graph neural network for predicting amino acid and nucleic acid positions (Jamali et al., 2024).

Here, we describe how to use our methods, ComplexModeler and CryoZeta (Zhang et al., 2026), for protein–DNA/RNA complex modeling on our web server (https://em.kiharalab.org/). ComplexModeler integrates CryoREAD and DiffModeler, developed by our group, for nucleic acid modeling and protein fitting, respectively. CryoREAD detects phosphate, sugar, and base positions to build the backbone and construct full atomic models (Wang et al., 2023). DiffModeler fits protein structures, including AlphaFold2 predictions (Jumper et al., 2021), into enhanced density maps by a deep neural network (diffusion model). CryoZeta uses a diffusion model‐based structure modeling pipeline, similar to AlphaFold3 (Abramson et al., 2024). It integrates cryo‐EM map density information into a diffusion model‐based structure modeling pipeline to generate protein–DNA/RNA complex structures (Zhang et al., 2026). CryoZeta shows substantially improved modeling performance over existing methods. Here we describe the protocols for the structure modeling of protein–DNA/RNA complexes using ComplexModeler and CryoZeta on the EMSuite webserver.

### EMSuite Server

The EMSuite server (https://em.kiharalab.org) is a web‐based platform by the Kihara Lab that makes advanced computational algorithms geared towards the structural biology community easily accessible to researchers. Many modern modeling methods, particularly deep learning approaches, require substantial CPU and GPU resources, limiting accessibility. Although the source code for all methods on the web server is available on GitHub (https://github.com/kiharalab/), installation and use still require a certain level of technical expertise. The EMSuite server addresses this barrier by hosting all required computational resources on the server side and allowing users to run resource‐intensive jobs by submitting using only a standard web browser.

The webserver's main page provides tutorials, a roadmap for available tools, and access to account registration. While account registration is not required to run a job, it is encouraged to help users track job progress, communicate with developers, and review past submissions to the job page. A notable aspect of the EMSuite server is its emphasis on user support. Each job submission's results page includes a feedback section where users can post questions about their jobs. These questions are typically rapidly addressed by the development team.

Available algorithms on the web server are organized in a roadmap that categorizes the available tools by input type, resolution range, and methodology. This helps users select the appropriate tools to use for their data. The server has six main categories of algorithms: structure modeling, structure validation, structure refinement, unknown protein finding, secondary structure detection, and structure fitting. In the structure modeling category, there are protein‐specific methods such as DeepMainmast (Terashi et al., 2024b) for maps at resolutions up to ∼5 Å, while DiffModeler (Wang et al., 2024) and DMcloud (Terashi et al., 2025) can accommodate lower resolution maps up to 15 Å through structure fitting approaches. As mentioned above, we have CryoREAD (Wang et al., 2023) for a DNA/RNA‐specific modeling method, while protein–DNA/RNA complex structures can be built with ComplexModeler and CryoZeta (Zhang et al., 2026), which are highlighted in this article. Structure models are validated with DAQ‐Score (Terashi et al., 2022), and DAQ‐Refine helps with structure refinement (Terashi et al., 2023). When protein sequences in a map are unknown, DAQFinder can be used for identifying them. Emap2sec (Maddhuri Venkata Subramaniya et al., 2019) is for protein secondary structure detection in a map, and Emap2sec+ (Wang et al., 2021) further extends it to also detect nucleic acids in a map. VESPER (Han et al., 2021) performs map‐to‐map and individual structure‐to‐map fittings.

Comprehensive tutorial pages are provided for each tool, including runnable examples, input file requirements, and step‐by‐step instructions. Collectively, EMSuite is designed to lower the technical barrier to using state‐of‐the‐art cryo‐EM modeling tools while maintaining broad accessibility for the structural biology community. We have released several tutorials of the EMSuite server and its tools in the past, including: book chapters on protein complex modeling tools (Park et al., 2026), protein modeling tools (Punuru et al., 2025), and protein secondary structure detection (Baghirov et al., 2025). In this article, we focus on two tools for protein–DNA/RNA complex modeling, which are not described in the earlier articles.

### Overview of the Software

Here we briefly explain the algorithms of ComplexModeler and CryoZeta. While both these methods can perform protein–nucleic acid complex structure modeling they have their strengths and limitations. ComplexModeler works better than CryoZeta for lower resolution maps (close to 10 Å resolution and worse) if accurate structural templates are available. It also works better for very large complexes given the protein modeling part is essentially structure fitting. However, its performance can be limited if there are no reliable model templates. Also, when the local resolution for DNA/RNA regions is between 5 and 10 Å, the resulting structure might be fragmented. On the other hand, CryoZeta is a new, more reliable method for de novo structure modeling, and will make accurate structures when the map resolution is up to ∼8 Å. But since it uses an end‐to‐end neural network, it has a size limit of ∼3000 total residues/nucleotides for modeling.

#### ComplexModeler

ComplexModeler functions as a two‐step pipeline to model protein–DNA/RNA complexes. It models protein chains and nucleic acid chains separately before assembling the final complex structure. The protein part is modeled by DiffModeler, while the nucleic acid part is modeled by CryoREAD.

The protein modeling component, DiffModeler, begins by processing the raw cryo‐EM density map to locate the protein backbones. It employs a diffusion model that has been trained to recognize the distinctive local density patterns that protein backbones produce to enhance and clarify the map, essentially cleaning up the signal to make backbone positions more legible. It then uses AlphaFold2 to generate predicted structures for each individual protein chain in the complex from the user‐provided amino acid sequence and then uses the structure fitting program VESPER to place these chains into the backbone‐enhanced map, generating 100 candidate orientations and positions for each subunit.

The final step is the selection of the combination of the fitted single chain protein poses to build the complete complex structure within the EM density map. Since checking every possible combination of poses would be computationally impractical, DiffModeler adopts a greedy assembly approach. In each step, it chooses the subunit pose with the best fit score and incorporates it into the model. The density corresponding to that subunit is then masked in the diffused backbone map, and any remaining poses that overlap with this masked region are removed. The process continues by selecting the next highest‐scoring pose that does not overlap, repeating iteratively. In this way, DiffModeler efficiently assembles large multi‐chain complexes while preventing steric clashes and conflicts in the density map.

For nucleic acid chains, CryoREAD is used. It identifies the positions of phosphate groups, sugars, and nucleotide bases and then traces and connects these into a complete atomic model of the DNA or RNA. First, at each grid position in the input cryo‐EM density map, the probability of that position being a phosphate, sugar, base, and the four base types is computed by a deep neural network with U‐Net architecture. Using these detected classes, the backbone of the nucleic acid is then traced. To do this tracing, the grid points that have class predictions are first clustered to find the representative nodes for each structure class predicted. The representative nodes for the prediction of the sugar class were then connected to form a graph, and the backbone is traced. Since we take in as input the sequence of the nucleic acids in the map, the next step is to assign the sequence to each of the sugar nodes. First, the traced backbone paths of the sugar nodes are cut into segments with a length of 20 nodes. These segments are then aligned with the user‐provided nucleic acid sequence to identify the top 20 sequence fragments, which are then assembled by maximizing the sum of the probability of the assigned bases with the sugar nodes. At the end of this step, we have a sugar backbone that has assigned bases from the sequence. To this structure, the phosphate and base nodes that satisfy certain distance conditions to the sugar node are added. To obtain the full atomic structure, the triangle of sugar, phosphate, and base nodes are superimposed with the standard conformation and then refined using Phenix (Liebschner et al., 2019) and COOT (Emsley et al., 2010).

**Figure 1:**
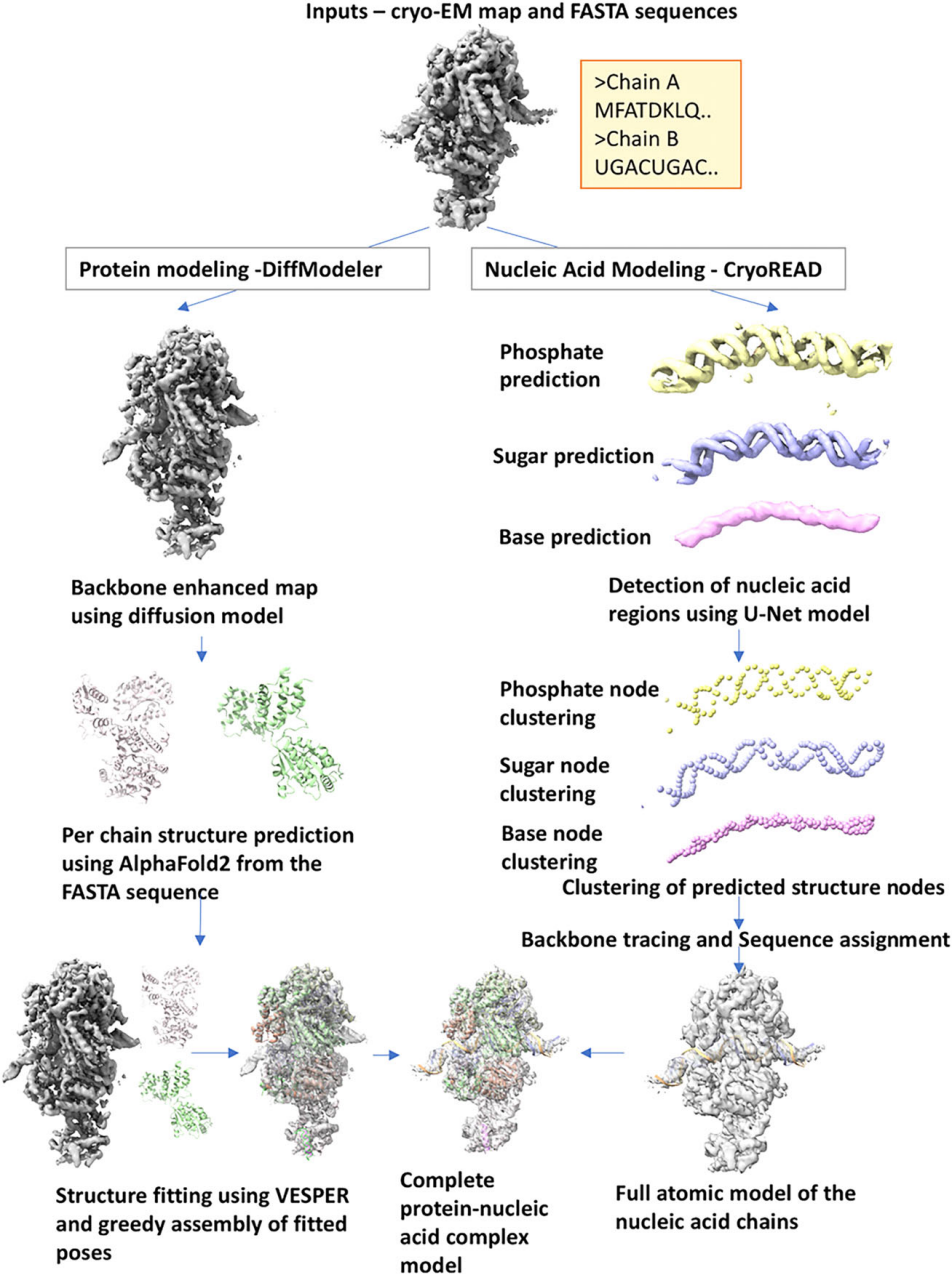
Overview of ComplexModeler. The steps for DiffModeler are shown in the flowchart on the left while the steps for CryoREAD are shown in the flowchart on the right. The overview shows the modeling of the protein components of Drosophila ORC bound to DNA (EMD ID: 22329, Resolution 3.9 Å) density map using DiffModeler and the DNA component using CryoREAD with the final output of the complete ComplexModeler pipeline being the assembled protein–nucleic acid complex.

Once both the protein and nucleic acid models are built independently, ComplexModeler combines them into a single protein–nucleic acid complex model (Fig. 1).

#### CryoZeta

CryoZeta takes one or more biological sequences (protein, DNA, and/or RNA) and a cryo‐EM density map as input. It then produces a full atom 3D model that is consistent with the EM density. It directly integrates spatial features extracted from a cryo‐EM density map into a diffusion model‐based structure generation pipeline, similar in concept to AlphaFold3. Instead of building a model first and then checking it against the cryo‐EM density map or separately fitting predicted structures into density, CryoZeta incorporates the map density information during the structure generation process itself, enabling the construction of accurate 3D structure complex models. The result is hence a unified pipeline that handles protein, DNA, and RNA all within the same framework, without needing separate special modules for each molecule type, and produces complex models consistent with the experimental density.

The input protein, DNA, and/or RNA sequences are first used to generate Multiple Sequence Alignments (MSAs). These MSAs are processed through the AlphaFold3 featurization pipeline to produce per‐residue single representations and pairwise residue‐residue representations, which encode evolutionary information.

Next, two separate 3D U‐Net networks operate on the input density map to extract information about the positions and identities of atoms and residues. The atom detection U‐Net classifies each voxel into one of eight atom classes (backbone heavy atoms, Cα, Cβ, and sidechain atoms for proteins and C1’, sugar, base, and phosphate atoms for nucleic acids). The second U‐Net network classifies voxels into 20 standard amino acids and four nucleotide base classes. The predicted Cα positions (for proteins) and C1’ positions (for nucleic acids) are clustered using the mean‐shift algorithm to yield a set of positional anchors called support points.

Subsequently, the EM‐Pairformer network operates on single‐residue representations, residue‐residue pair representations, support point‐residue pair representations, and support point‐support point representations. These 4 representations are iteratively updated during the training process. At each step, the sequence‐derived representations interact with the density‐derived support point information, ensuring the final learned representations are jointly consistent with both the sequence context and the spatial density constraints derived from the EM map. Conceptually, the EM‐Pairformer examines correspondence between identified atom points in the EM map and amino acid residues (or nucleotides) in the input sequences.

**Figure 2:**
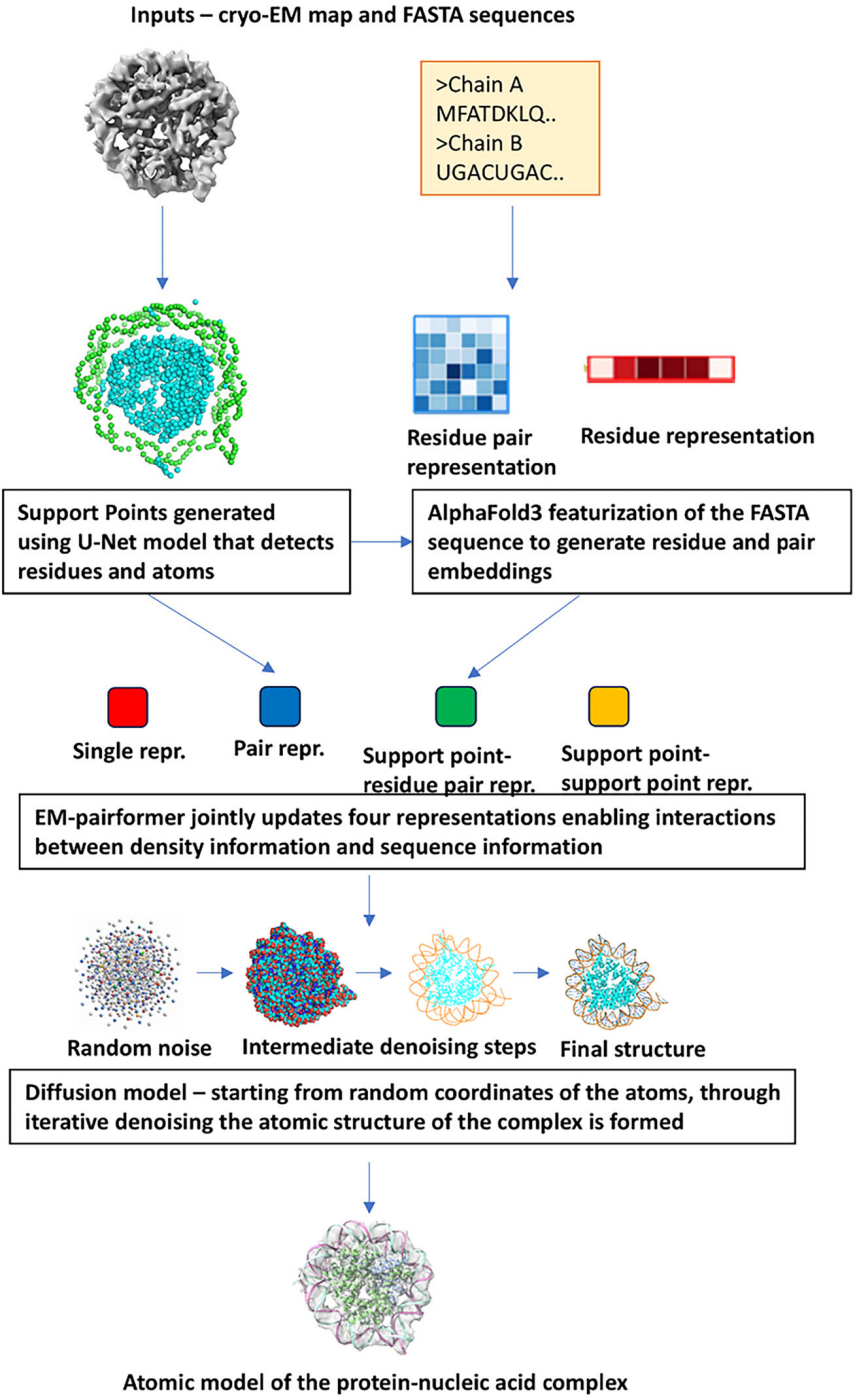
Overview of the CryoZeta pipeline. CryoZeta takes as input the cryo‐EM map and FASTA sequences of the protein and DNA/RNA chains. It processes the density map to generate support points, further the FASTA sequence is processed using the AlphaFold3 featurization pipeline to produce representations that encode evolutionary information. The support points and the sequence features are then passed into an EM‐Pairformer where the joint representation of the EM map and sequences are learned. The final learned representations are then passed into a diffusion module that produces the final atomic model of the complex (EMD‐3949, Resolution 5.1 Å).

The updated single and residue‐residue pair representations from the EM‐Pairformer are then passed to a diffusion‐based structure generation module. This module generates full 3D atomic structure by iteratively denoising and refining atomic positions starting from Gaussian noise in 3D coordinate space guided by the learned representations. Because the representations encode density‐derived spatial constraints from the EM‐Pairformer, the generated structure is inherently biased toward conformations consistent with the density map (Fig. 2).

CryoZeta was shown to perform substantially better in modeling than existing methods in benchmark studies on protein complexes and protein–DNA/RNA complexes (Zhang et al., 2026).

## Basic Protocol 1 PROTEIN–NUCLEIC ACID STRUCTURE MODELING USING ComplexModeler ON THE EMSuite SERVER

To run ComplexModeler on the EMSuite server, users only need to upload a cryo‐EM map and sequence data with some parameter values. Then the server will run the ComplexModeler pipeline and return the results to users. Below is a step‐by‐step guide on the details of job submission and result retrieval on the web server.

### Necessary Resources

#### Hardware

A standard laptop or desktop computer with access to a web browser

##### Software

None required; all jobs are submitted through a standard web browser

##### Files

Input data:
  Protein and nucleic acid sequences provided in FASTA format
  Cryo‐EM map into which the user wants to model the structure

#### Steps to submit ComplexModeler jobs

**Figure 3:**
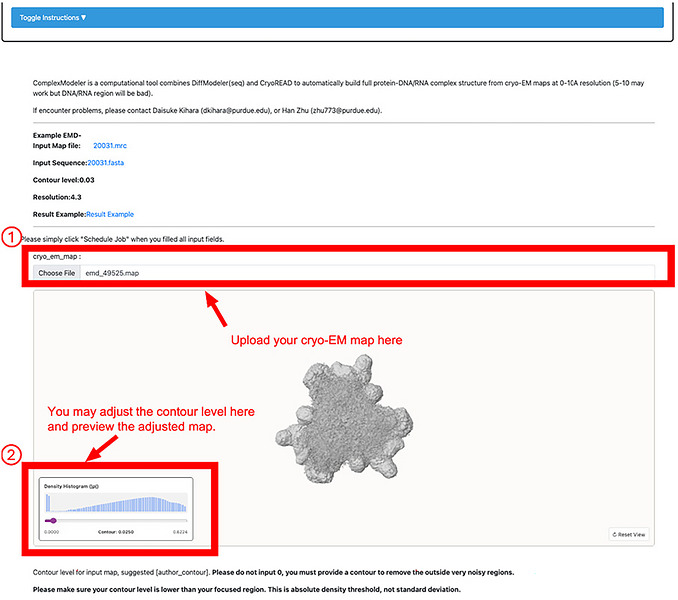
ComplexModeler input page. (**1**) Button for uploading your cryo‐EM map file. (**2**) A sliding bar to adjust the contour level to show the density distribution of the uploaded map.

1 Access the job submission page: Navigate to the EMSuite server. Select ComplexModeler from the “Algorithm” tab on the top menu. It is located at: https://em.kiharalab.org/algorithm/ComplexModeler.
2 Select a cryo‐EM map to upload: Click on the “Choose File” button to select the map file on your local computer and upload the input cryo‐EM map file in the MRC or MAP format (Fig. 3).

**Figure 4:**
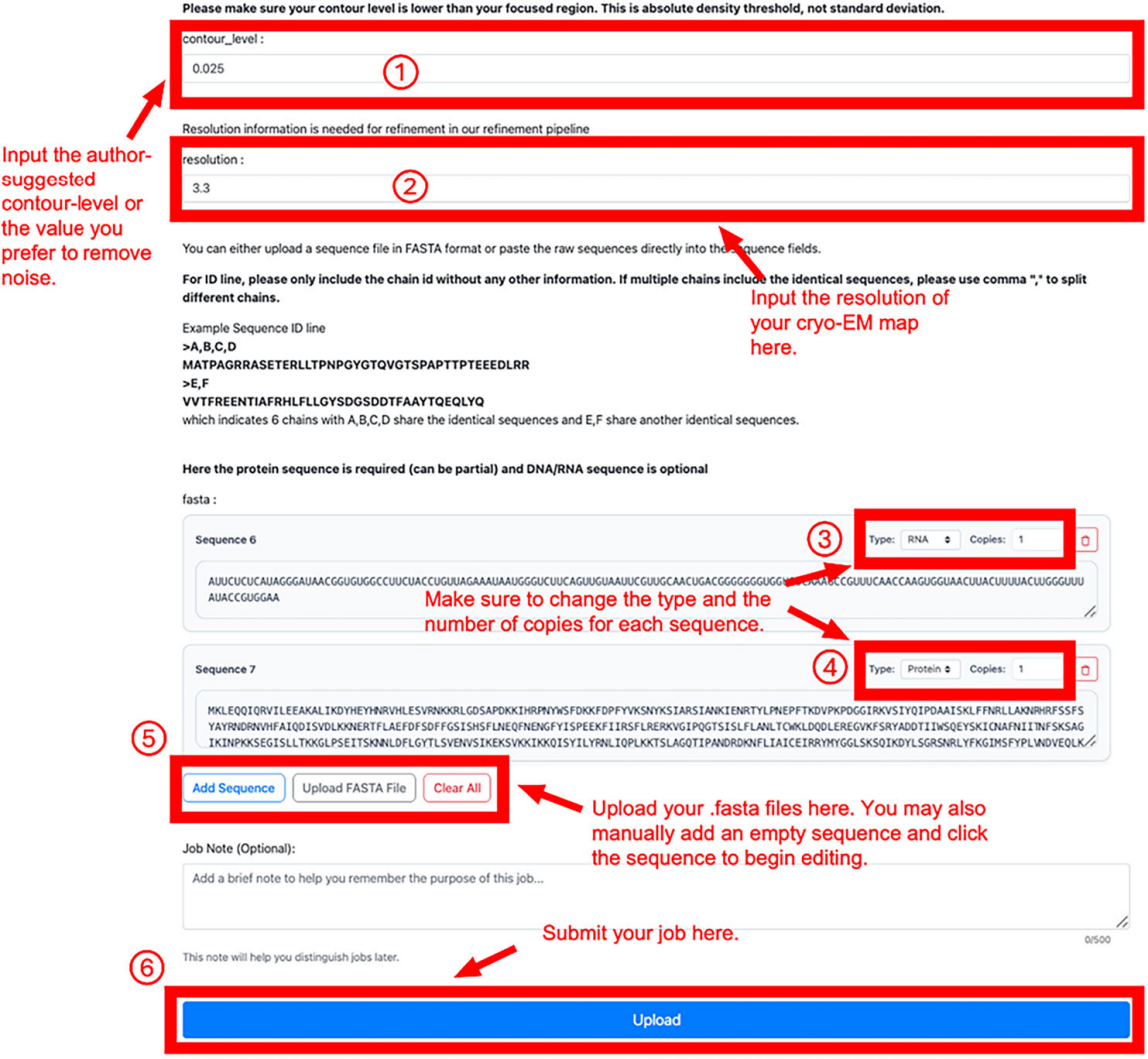
ComplexModeler input page describing various input fields. (**1**) The input box for contour level. (**2**) The input box for the map's global resolution. (**3**‐**4**) Buttons for choosing the type and number of copies for your uploaded sequence. (**5**) Button for uploading FASTA sequence file. (**6**) Button for job submission.

3 Set the contour level for the map (Fig. 4). This is used to remove the noise regions in the map. Users can determine the contour level by adjusting the level in 3D views such as UCSF ChimeraX (Meng et al., 2023), or preview the adjusted map using the embedded 3D viewer on the webpage (Fig. 3).
  We recommend setting the contour level to show the main density while masking out the noise regions. If the cryo‐EM map is from EMDB (https://www.ebi.ac.uk/emdb/), we recommend that users set the contour level to half of the author‐recommended value.

**Figure 5:**
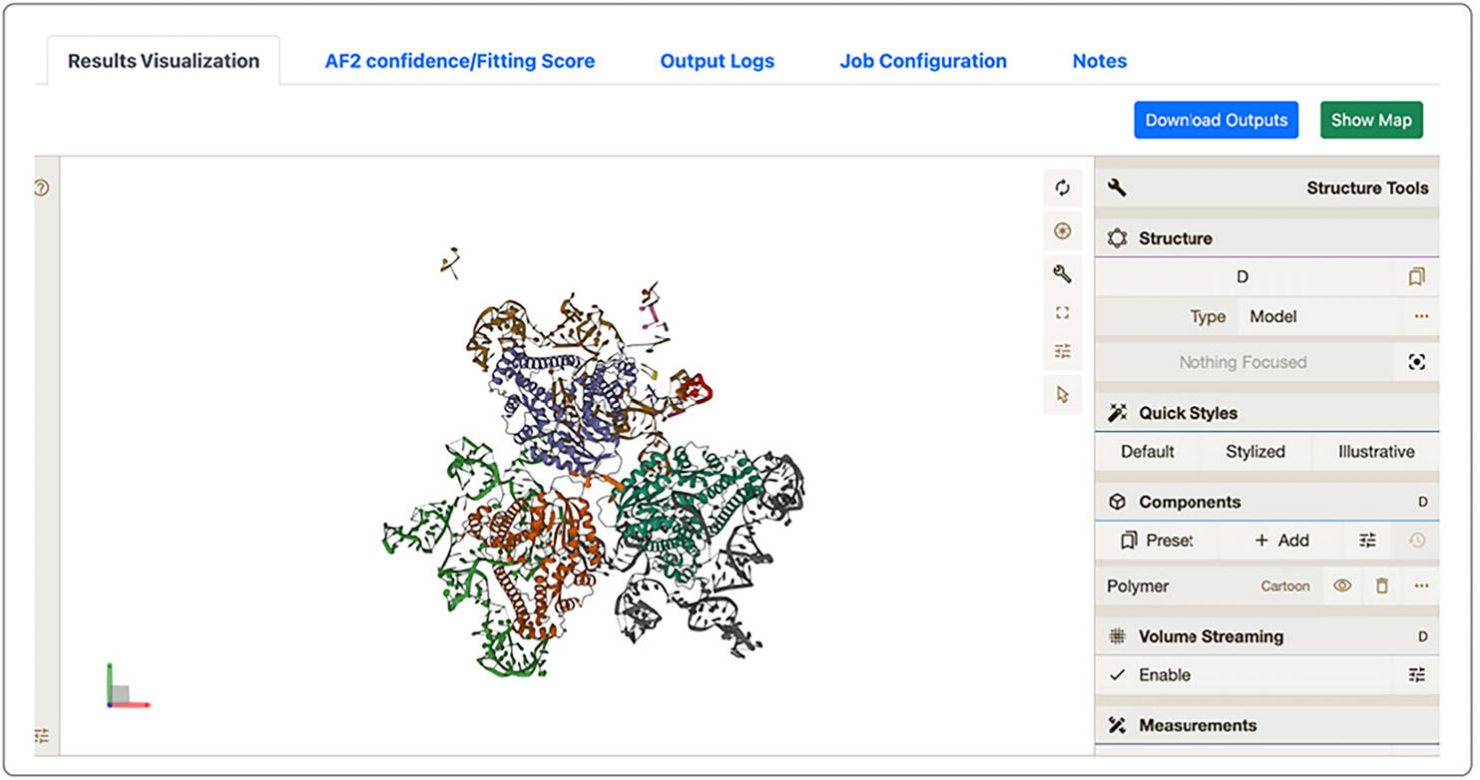
Visualization page of ComplexModeler results. The Structure Tools on the right of the viewport allow the users to modify the display style of the structure and map. Users can also focus on certain regions of the structure as well as measure distances and angles between different regions of the structure. The structure shown in the current image is PDB 9NLX, EMDB ID: 49525.

4 Set the approximate resolution (Fig. 4). If the resolution is high (e.g., <2.0 Å), backbone enhancement by the diffusion model will be skipped. Resolution is also used to guide the final refinement in the pipeline.
5 Select the sequence file for the target proteins to upload. Users can upload the sequence data in the FASTA format by clicking on the “Choose File” button (Fig. 4). Users can also edit the sequence data on the webpage by clicking the “Edit” button after uploading. The protein sequence is required, and the nucleic acid sequence is optional. Each sequence entry should have an ID line and a sequence line. The ID line should start with “>” followed only by the sequence ID without any other information. For example: “>A, B, C”.
  The sequence is used to search for templates in Protein Data Bank (PDB) and AlphaFold Database (AFDB), which are used to build the models. Input sequences are searched by BLASTP against the PDB95 and AlphaFold Database. For the PDB95 search, only hits with an E‐value of 0 are considered, and the first hit whose chain length falls within 90% to 110% of the query is selected as the template. If no hit satisfies these criteria, the search is performed against the combined PDB + AlphaFold Database, where the best (by E‐value) length‐compatible hit is selected from the top 500 results.
6 Inspect the resulting model interactively (Fig. 5).

It provides an interactive 3D viewer to visualize the predicted structure models. The input map can also be visualized by clicking the “Show Map” button on the upper‐right corner of the viewer. The output models in CIF format can be downloaded by clicking the “Download Outputs” button. On the right side of the viewport, multiple options are available for changing the display style of the structure, including default, stylized, and illustrative modes. Users can also display measurements such as distances, angles, and planes between selected atoms. Different map views, including bounded box, whole structure, and around‐camera views, can also be explored. The “AF2 Confidence/Fitting Score” tab allows users to color the displayed structure using pLDDT values from AlphaFold2‐predicted chain models. The “Output Logs” tab provides program execution details for the job, while the “Job Configuration” tab displays the user‐defined input parameters. The “Notes” section allows users to record private notes for future reference.

#### Case study of structure modeling by ComplexModeler

In this example, we describe the structure modeling of the trimeric SenDRT9 RT–ncRNA complex from *Salmonella enterica* using ComplexModeler. The input map (EMDB ID: EMD‐49525) has a global resolution of 3.2 Å. The complex is a homotrimer consisting of three protein subunits of 499 amino acids each and three RNA molecules of 147 nucleotides each. It took ∼5 hr to model this complex on the web server.

**Figure 6:**
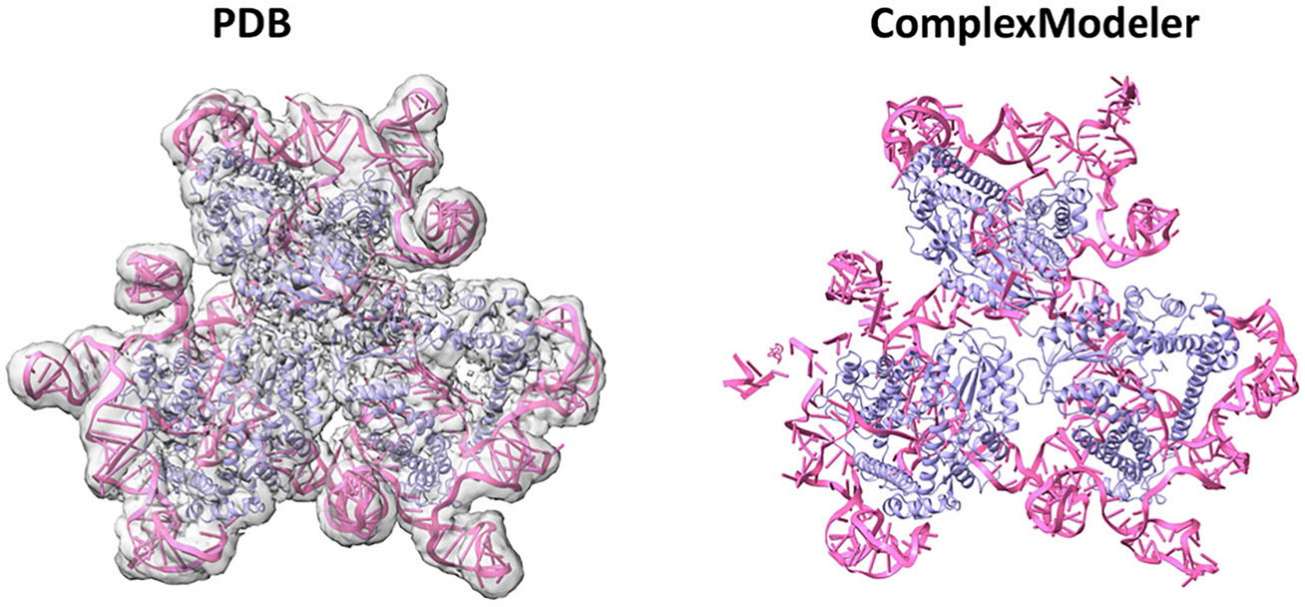
Example of a model built by ComplexModeler. The predicted structure of the trimeric SenDRT9 RT‐ncRNA complex structure in comparison with the corresponding PDB entry. (PDB ID: 9NLX; EMD‐49525, Resolution: 3.2 Å). The proteins are shown in purple while the nucleic acid regions are in pink

Figure 6 shows the ComplexModeler model with the structure in PDB (PDB ID: 9NLX). The agreement between the modeled structure and the native structure was calculated using a TM‐score. A TM‐score assesses the global similarity between two macromolecular structures. The model has a TM‐score (which ranges from 0 to 1, with 1 for an identical structure) of 0.79 and a root mean square deviation (RMSD) of 2.64 Å to the PDB entry. We provided the input map for modeling at half the author‐recommended contour level, a level that we commonly use in our lab for modeling tasks. In this model, the protein structures have a TM‐score of 0.97, while the RNAs have a mediocre TM‐score of 0.53. The overall conformation of RNAs was captured in the model; however, some nucleotides were not modeled, as seen in the left region of the ComplexModeler model. This leads to a fragmented chain of nucleic acids in that region, which is reflected in the TM‐score. This as a limitation of CryoREAD, which can occur when the local map resolution is insufficient. In such cases, users may need to perform additional structure refinement starting from the generated model.

## Basic Protocol 2 PROTEIN–NUCLEIC ACID STRUCTURE MODELING USING CryoZeta ON THE EMSuite SERVER

CryoZeta is a new tool available in the EMSuite server that performs end‐to‐end structure modeling of macromolecular complexes. Below, we provide a step‐by‐step guide for submitting jobs on the web server. The procedure is similar to that used for ComplexModeler.

### Necessary Resources

#### Hardware

A standard laptop or desktop computer with access to a web browser

##### Software

None required; all jobs are submitted through a standard web browser

##### Files

Input data:
  Protein and nucleic acid sequences provided in FASTA format
  Cryo‐EM map into which the user wants to model the structure

#### Steps to submit CryoZeta jobs

**Figure 7:**
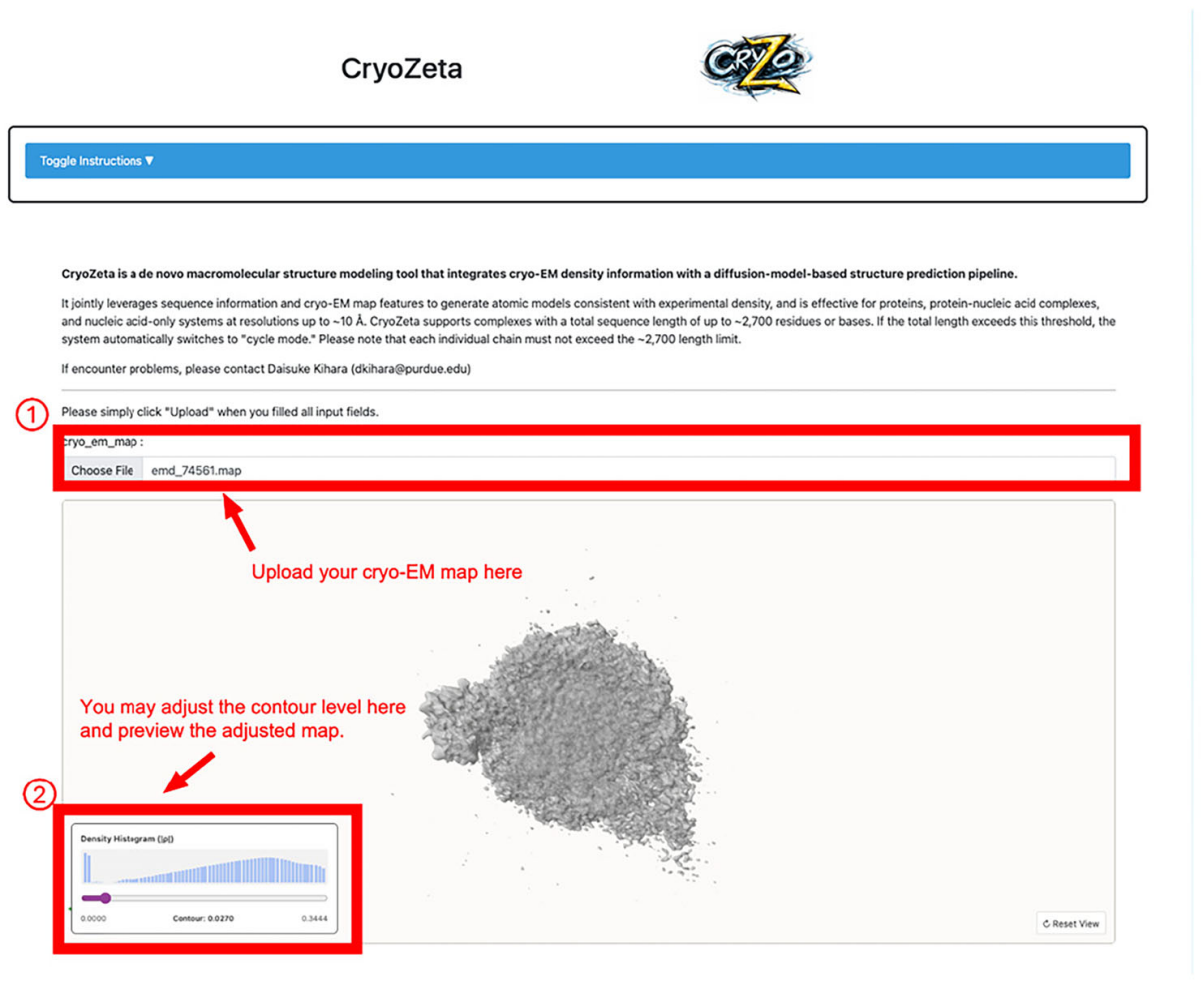
CryoZeta input page. The red box on the top shows the window to upload an input EM map. The box at the bottom provides a sliding control for adjusting the map contour level. Users can use this tool to determine an appropriate contour level for input into the parameter window. (**1**) Button for uploading a cryo‐EM map file. (**2**) A sliding bar to adjust the contour level.

1 Access the job submission page: Navigate to the EMSuite server. Select CryoZeta from the “Algorithm” tab on the top menu. It is located at: https://em.kiharalab.org/algorithm/CryoZeta.
2 Upload a cryo‐EM map: Click on the “Choose File” button to select and update the input cryo‐EM map file in the MRC or MAP format (Fig. 7).

**Figure 8:**
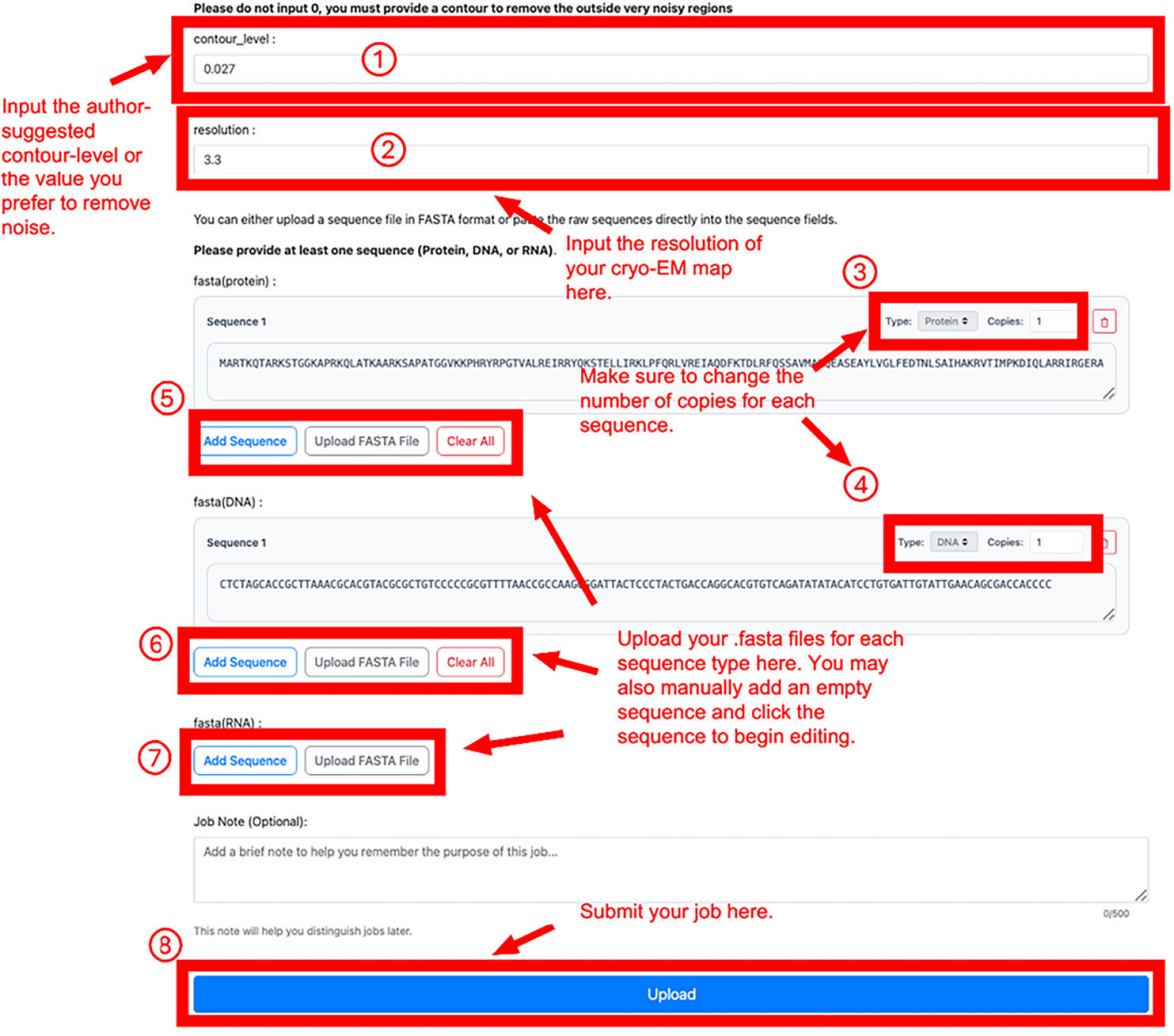
The parameter input page for CryoZeta. (**1**) The input box for contour level. (**2**) The input box for the map's global resolution. (**3**‐**4**) Buttons for choosing the type and number of copies for your uploaded sequence. (**5**‐**7**) Buttons for uploading FASTA sequence files for protein, DNA, and RNA, respectively. (**8**) Button for job submission.

3 Next, input several parameters of the map (Fig. 8). The first parameter is the contour level of the map. Similar to ComplexModeler, the contour level can be determined by the sliding window implemented in the web page (Fig. 7).
  We recommend setting the contour level to show the main density while masking out the noise regions. If the cryo‐EM map is from EMDB (https://www.ebi.ac.uk/emdb/), we recommend users to set the contour level to half of the author‐recommended value.

**Figure 9:**
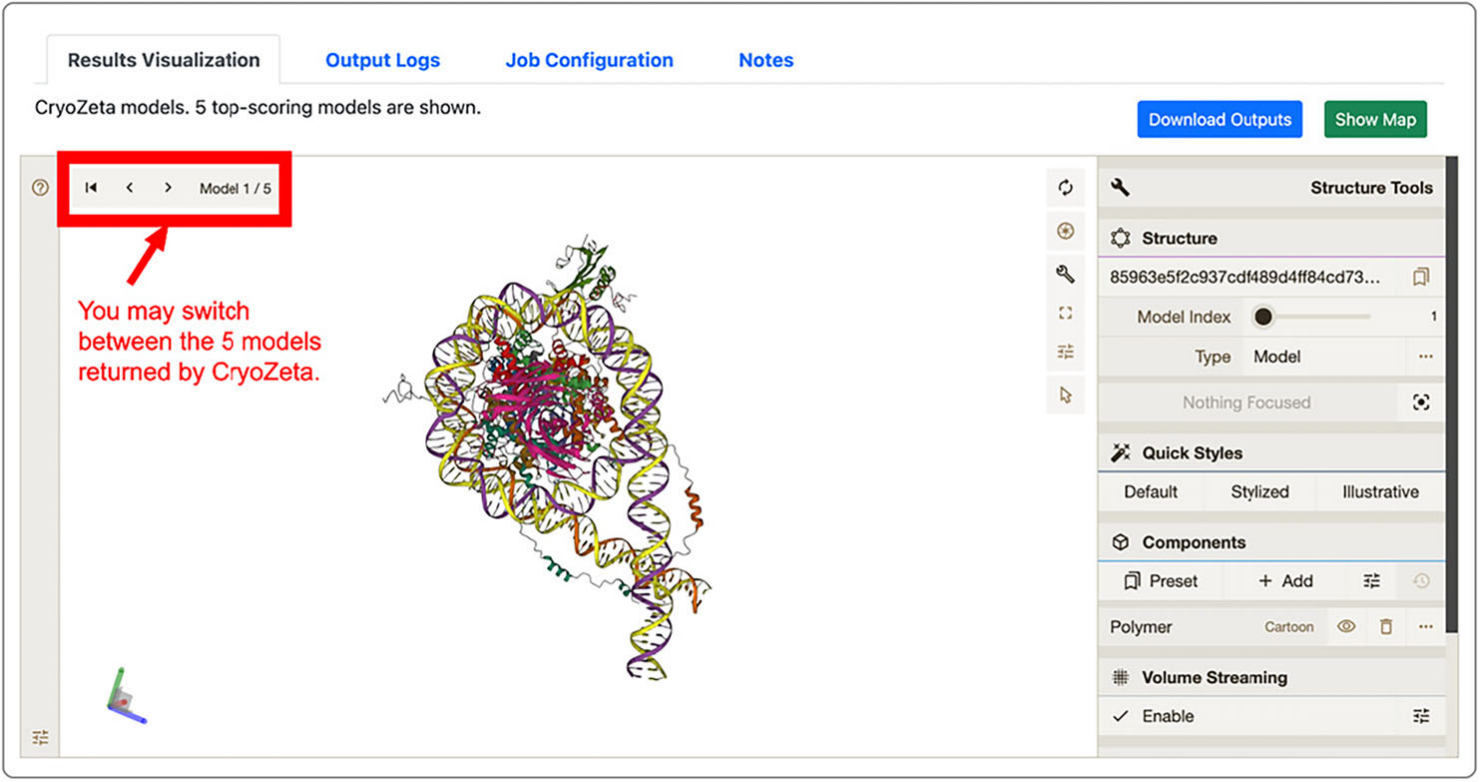
Visualization page for CryoZeta structure modeling results. Users can switch among the five predicted models by clicking the buttons in the red box. By default, the first model, corresponding to the highest model reliability score, is displayed. The structure shown in the viewport is PDB 9ZQ9 with EMDB ID: EMD‐74561.

4 Next, specify the approximate resolution of the map (Fig. 8). This information guides how the model interprets the density map and is used in two ways. First, atom detection in the EM map is performed differently for high‐ and low‐resolution maps, using a cutoff of 4.5 Å. Second, the resolution is used when computing the cross‐correlation between the structure model and the map, which contributes to the model confidence score used for model selection.
5 Upload protein, DNA, and RNA sequence data for the target complex to model. Users can upload the sequence data in the FASTA format by clicking on the “Choose File” button (Fig. 8). Users can also edit the sequence data on the webpage by clicking the “Edit” button after uploading (Fig. 8). If the target is a homomultimer, the sequence should be repeated for each subunit. For example, if the target is a homotrimer, there should be three identical sequence entries in the FASTA file.
6 Inspect the resulting model interactively (Fig. 9).
  The server provides an interactive 3D viewer for the predicted model. The input map can also be visualized by clicking the “Show Map” button on the upper‐right corner of the viewer. The output is a zip file containing the 5 top predictions ranked by model reliability score of CryoZeta. It is a combination of Cα recall (the fraction of Cα atoms in the model that are closely placed to predicted Cα positions), cross correlation of the simulated map of the model and the uploaded map, and a penalty for large Cα‐Cα distances, which are typically caused by incorrect domain connections. The predicted models are in the CIF format.

#### Case study of modeling by CryoZeta

In this example, we modeled the structure of the nucleosome with an SSB at SHL –2.8 in complex with the WGR domain of human PARP2. The input map (EMD‐74561, 3.3 Å) was obtained from EMDB, and the corresponding sequence data were obtained from RCSB PDB (PDB ID: 9ZQ9). It consists of a protein of 11 chains with 1649 amino acids in total and 3 DNA chains with 394 nucleotides in total. The modeling took ∼16 hr using these inputs.

**Figure 10:**
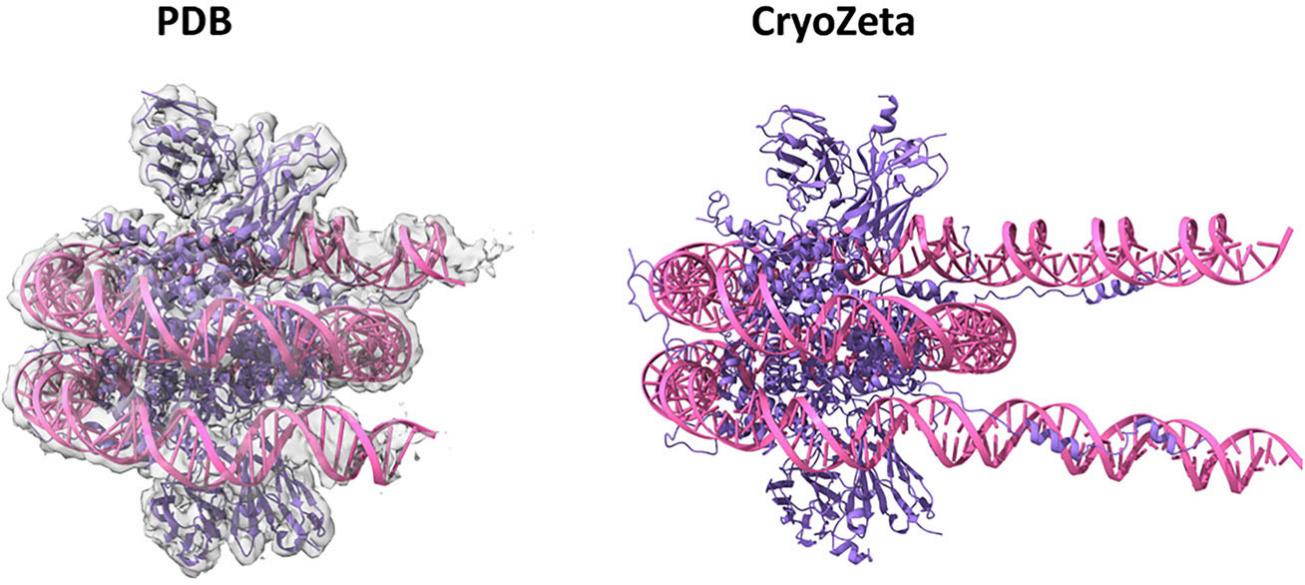
Example of a model built by CryoZeta. The PDB structure and a model built by CryoZeta for the nucleosome in complex with WGR domain of human PARP2. EMDB ID: 74561, PDB ID: 9ZQ9. The map resolution is 3.30 Å. The proteins are shown in purple, and DNA is shown in pink.

The predicted protein structure has an overall RMSD of 2.31 Å to the native structure, while the predicted DNA structure has an RMSD of 0.77 Å. The TM‐scores for both the protein and DNA regions are 0.98. Figure 10 compares the experimental structure of the nucleosome in complex with the WGR domain of human PARP2 and the corresponding CryoZeta model generated from the sequences and input density map. For CryoZeta modeling, we provided the full‐length protein and DNA sequences, including regions not modeled in the deposited PDB structure. These unmodeled DNA regions were predicted as extended segments beyond the deposited model. Additionally, protein regions were also modeled as extended helices near the extended DNA regions. Upon lowering the map contour level, weaker density corresponding to the extended DNA region becomes visible, providing support for the CryoZeta model.

## COMMENTARY

### Critical Parameters

To obtain robust results from Basic Protocols 1 and 2, the quality of the input map being within the listed resolution ranges is critical. ComplexModeler would work for maps up to ∼10 Å resolution while modeling for CryoZeta is expected to be highly successful for maps up to ∼8 Å. Additionally, providing the FASTA sequence that pertains to the regions present in the input map and avoiding providing sequences for regions not present in the map is essential.

### Troubleshooting

Table 1 highlights a troubleshooting guide for some common problems along with their causes and solutions.

**Table 1:** Causes and Solutions to Possible Problems With Basic Protocols 1 and 2

Problem	Possible cause	Solution
ComplexModeler results in fragmented or incomplete chains for nucleic acid regions	CryoREAD component of the algorithm may be unable to effectively model the nucleic acid regions if the local map resolution is not good	Use the ComplexModeler output as a starting point for downstream manual refinement
No remaining density in the input map after contouring for both ComplexModeler and CryoZeta	Contour level provided by the user is too high	Adjust the contour level to ensure noise removal but not the removal of regions of interest
Unable to model due to sequence size limitations for CryoZeta	The algorithms have an upper limit on the number of residues or nucleotides it can handle due to computational requirements	Segment the map into a smaller region and provide the sequence that corresponds to the local region
Input map too big, e.g., viral capsid maps for example for CryoZeta	Large maps can lead to out of memory issues on the computational hardware	Segmenting regions of interest instead of providing the full map

### Understanding Results

The output for both the protocols is the atomic coordinates of the protein and nucleic acid structures modeled for provided sequences and the cryo‐EM maps. These results can be visualized in the “Results Visualization” tab on the webserver or can also be downloaded for further analysis. Downloaded structure files can be visualized by Pymol, ChimeraX, or other commonly used biological structure viewing software.

### Time Considerations

The runtime of both protocols depends on the size of the input cryo‐EM map, the length of the input FASTA sequences, and the current workload of the server. In general, the modeling process takes from 4 to 24 hr. When the server is heavily utilized, jobs may spend additional time in the queue waiting for computational resources to become available before execution begins. For the case studies presented above, ComplexModeler required ∼5 hr to model a homotrimeric complex consisting of 499 amino acids and 147 nucleotides per chain. CryoZeta required ∼16 hr to model a complex containing a total of 1649 amino acids and 394 nucleotides.

### Summary

This article describes protocols for obtaining protein–DNA/RNA complex structures from cryo‐EM density maps using ComplexModeler and CryoZeta on the EMSuite web server. ComplexModeler integrates DiffModeler (for protein structure modeling) and CryoREAD (for nucleic acid modeling) to construct complete complex structures. CryoZeta is an end‐to‐end modeling method based on the AlphaFold3 network architecture that incorporates cryo‐EM density information into the structure prediction pipeline.

Although this article focuses on protein–DNA/RNA complex modeling, EMSuite also provides multiple additional tools, as highlighted in the EMSuite section. The website includes a roadmap describing suitable applications of these tools across different resolution ranges and macromolecule types. Tutorials for using these tools are also available on our lab's YouTube channel at: https://www.youtube.com/@kiharabioinformaticslab2452.

Together, these resources are intended to make advanced cryo‐EM modeling methods more accessible to the broader structural biology community. We expect EMSuite to facilitate efficient modeling and validation of increasingly complex macromolecular assemblies derived from cryo‐EM experiments.

### Author Contributions

**Anika Jain**: Data curation; investigation; validation; visualization; writing—original draft. **Kefan Cao**: Data curation; investigation; validation; visualization; writing—original draft. **Daisuke Kihara**: Conceptualization; funding acquisition; project administration; resources; supervision; writing—review and editing.

### Conflict of Interest

The authors have no conflicts of interest to declare.

## Data Availability

Data sharing not applicable to this article as no datasets were generated or analyzed during the current study.
